# Selected adjunct cultures remarkably increase the content of bioactive peptides in Bulgarian white brined cheese

**DOI:** 10.1080/13102818.2014.969918

**Published:** 2014-10-28

**Authors:** Zhechko Dimitrov, Elena Chorbadjiyska, Irina Gotova, Kalinka Pashova, Svetla Ilieva

**Affiliations:** ^a^ R&D Center, LB-Bulgaricum PLC., Sofia, Bulgaria; ^b^ Department of Biotechnology, Faculty of Biology, Sofia University “St. Kliment Ohridski”, Sofia, Bulgaria

**Keywords:** ACE-inhibition, calcium-binding, anti-hypertensive peptides

## Abstract

Some lactic acid bacteria strains in milk media are capable of releasing bioactive peptides. In this study, we evaluated the angiotensin-converting enzyme (ACE)inhibitory activity of 180 lactic acid bacteria and selected several *Lactobacillus helveticus*, *L. delbrueckii* subsp*. bulgaricus* and *L. casei* strains that demonstrated strong ACE-inhibitory activity. The aim was to carry out a molecular study on the bioactive peptides released by the strains with the best ACE-inhibitory properties and by the strains demonstrating a calcium-binding effect. To the best of our knowledge, this is the first study of bioactive peptides in Bulgarian white cheese. Peptides with the strongest ACE-inhibitory activity were purified and sequenced. The strains were assessed for production of peptides with calcium-binding properties. These peptides were isolated, purified and sequenced. Two strains releasing bioactive peptides with the strongest ACE-inhibitory and calcium-binding activities were selected for development of cheese starters. The strain with the best ACE-inhibitory activity was *L. helveticus* A1, which releases the peptide Ala-Leu-Pro-Met as a main contributor to the ACE inhibition. The strain with the best calcium-binding activity was *L. casei* C3 releasing the peptide SpLSpSpSpE (fraction 15–20 of ß-casein) as a main contributor to calcium binding. After pilot production of cheeses with the developed starters, the ACE-inhibitory and calcium-binding effects were confirmed during the cheese ripening. The addition of the two selected adjunct cultures led to increased production of bioactive peptides in the cheese. In this way, it is possible to increase the functional properties of Bulgarian white brined cheese.

## Introduction

Milk proteins are precursors of many biologically active peptides which are inactive within the sequence of the precursor proteins but can be released by proteolysis during milk fermentation. Milk protein-derived bioactive peptides may function as exogenous regulatory substances with hormone-like activity on the different intestinal and peripheral target sites of the mammalian organism.[1] To date, anti-hypertensive peptides, together with phosphopeptides and immunomodulating peptides, are the favourite bioactive peptides for application to foodstuffs formulated to prove specific health benefits.[2,3] Bioactive peptides are short chains of amino acids which are released by the action of the enzymes of strains in the fermented products, but also those of the digestive system. There are four main fields in which the observed effect of consuming dairy products can be attributed to functional peptides: the digestive system, the body's defences (anti-microbial and immunomodulatory effects), the cardiovascular system (anti-hypertensive and anti-thrombotic effects) and the nervous system (oppoid peptides). Another important functional effect is bone strengthening through improvement of mineral absorption.[4] Angiotensin-converting enzyme (ACE; EC 3.4.15.1) has been associated with the renin-angiotensin system which regulates peripheral blood pressure, where it catalyses both the production of the vasoconstrictor angiotensin II and the inactivation of the vasodilator bradykinin. ACE inhibition results mainly in an anti-hypertensive effect but may also influence different regulatory systems involved in modulating blood pressure, immune defence and nervous system activity.[3,5] Several anti-hypertensive peptides that inhibit ACE have been isolated from milk products, and the ACE-inhibition activity of these peptides has been determined. The relation between ACE-inhibitory peptides and the chemical structure has not been confirmed, but it has been suggested that peptides with hydrophobic amino acids at the C-terminal position could be the most likely ACE inhibitors.[6] Peptides derived from casein by *L. helveticus* proteases have been shown to have ACE-inhibitory activities.[7] ACE-inhibitory activity of the casein-derived tripeptides Ile-Pro-Pro and Val-Pro-Pro has been proved *in vitro*.[8,9] Some bioactive peptides could have a potential effect on the bone accretion by improvement of the absorption of calcium and magnesium through the intestinal barrier. These are mainly phosphopeptides with high binding capacity to calcium and magnesium.[10] It was reported that probiotic yogurt containing strains of *L. casei*, *L. reuterii* and *L. gasseri* increased apparent calcium absorption bone mineral content in growing rats.[11] It is noteworthy that the ability of lactic acid bacteria to release bioactive peptides is strain dependent. That is why it is necessary to examine a large number of strains in order to find significant producers of peptides with functional properties.

In this study, we analysed a large number of lactic acid bacteria strains for their ability to inhibit ACE and for their Ca-binding capacity. The main goal of this study was to characterize the bioactive peptides released by the strains with the best ACE-inhibitory properties and calcium-binding effect. To the best of our knowledge, this is the first report on characterization of ACE-inhibitory peptides and Ca-binding peptides in Bulgarian cheeses. We studied the influence of selected strains with ACE-inhibitory and Ca-binding properties included in the starter on the release of functional peptides with such properties during cheese ripening.

## Materials and methods

### Assay of total ACE-inhibitory activity

ACE activity was determined by the method of Cushman and Cheung [12] modified by Nakamura et al. [13] with some additional modifications. After the fermentation of milk with each evaluated strain, the supernatant (5 mL, pH 4.3) was subjected to purification through a reverse-phase cartridge (Waters C18ec). The samples were then washed with water and the peptides were eluted with 5 mL 60% acetonitrile in 0.1% trifluoroacetic acid (TFA). The eluate was freeze dried and reconstituted in 1 mL 0.1% TFA. The substrate Hip-His-Leu was dissolved in 100 mmol/L of Na-borate buffer (pH 8.3) to a concentration of 6 mmol/L in the assay mixture. The final concentration of NaCl was 300 mmol/L. To 190 μL of substrate solution, 20 μL of purified supernatant or peptide fraction were added and the reaction was initiated by 40 μL of ACE enzyme solution (0.1 U/mL). The duration of the reaction was 30 min at 37 °C until stopping with 100 μL of 4 mol/L HCl. The extraction of liberated hipuric acid was performed with 1 mL ethyl acetate. After evaporation of the extragent and reconstitution with 1 mL of water, the concentration of hipuric acid was determined at 228 nm. The per cent of inhibition was calculated by the formula:
(1) 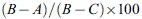
where *A* is optical density in both ACE and the peptide fraction, *B* is optical density without the peptide fraction, and *C* is optical density without ACE. The concentration of an ACE inhibitor needed to inhibit 50% of ACE activity is defined as the 50% inhibitory concentration (IC_50_).

### Purification and sequencing of ACE-inhibitory peptides

The supernatants were partially purified on reverse-phase cartridges as previously explained and subjected to centrifugal ultrafiltration with a 5000 Da membrane. TFA was added to the samples to 0.1% concentration and 1 mL was injected onto a reverse phase high pressure liquid chromatography (RP-HPLC) column Nucleosil C18. The gradient from 0% to 80% acetonitrile in 0.1% TFA was conducted for 45 min. Peptides were detected at 210 nm by means of a UV-detector (Shimadzu) and 1 mL fractions were collected. These fractions were freeze dried and evaluated for anti-ACE activity. The fractions demonstrating the highest ACE-inhibitory activity were run on high pressure liquid chromatography (HPLC) column Nucleodur Sphinx at specific gradient conditions for the respective fractions. For instance, the fraction from *L. helveticus* A1 was analysed at a 25%–35% acetonitrile gradient, and the fraction from *L. casei* C3, at 35%–45%. The last purification step was performed by means of ion-exchange HPLC column Shimadzu SCX containing benzene–sulfonic cation exchange groups in Li-mode. The elution was performed at a gradient of pH 3.0 to 9.0 in citrate buffers. Finally, single peaks with ACE-inhibitory activities were collected from the three strains that demonstrated the highest total ACE-inhibitory effect. Before sequencing, the amino acid content of the purified ACE-inhibitory peptides was determined after hydrolysis with 4 mol/L methanesulphonic acid and derivatization with phenyl-isothiocianate. Initial step for the sequencing was the fixing of the C-end of peptides to arylamine-polyvinyl-difluoride membrane, following the manufacturer's instructions (Millipore, USA). The sequencing from the N-end of the peptides was performed according to Edman et al. [14]

### Determination of total Ca-binding activity through an ion-selective electrode

The samples were deproteinized and demineralized as described above. The purified samples were buffered with Tris-HCl, pH 8.0, to a final concentration of 50 mmol/L and 100 μL of 0.1 mol/L CaCl_2_ was added to 10 mL of samples. After mixing for 1 min, the Ca^2+^ concentration was measured by means of a Ca^2+^ selective electrode (Orion 93-20, Boston MA) connected to an Orion 290A ion analyser. The calibration curve (mV/C^2+^) was built with standard Ca^2+^ solutions. The calcium bonded by the peptides was determined after subtraction of the measured free calcium from the total calcium.

### Purification of Ca-binding peptides from cultures with the highest Ca-binding activity

The supernatants from the 10 strains with the highest Ca-binding activity were ultrafiltrated through a 5000 Da membrane and subsequently loaded onto RP-cartridges Chromabond HLB. After washing with water, the peptides were eluted with 60% acetonitrile in 0.1% TFA. After evaporation of the eluent, the substances were reconstituted in 1 mL of 10% acetonitrile in 0.1% TFA. The solution was injected onto RP-HPLC column Hypersil peptide (Macherey-Nagel). Using a Shimadzu HPLC system, 1 mL fractions were collected. The eluents were: eluent 1 (0.1% TFA in water) and eluent 2 (80% acetonitrile in 0.1% TFA). The gradient of eluent 2 was from 2% to 60% for 45 min at 40 °C and a flow rate of 1 mL/min. The fractions with the best Ca-binding activity were fractionated additionally using a BioBasic-300 cation-exchange column. The eluents used were: eluent A (20 mmol/L KH_2_PO4, pH 4.8) and eluent B (NaAcetate in 25% acetonitrile) at 40 °C and a flow rate of 1 mL/min. The gradient of eluent B was from 0% to 60% for 15 min. All fractions collected after ion-exchange purification were evaluated for their Ca-binding activity. The fractions with the best Ca-binding activity were freeze dried and subsequently the corresponding peptides were sequenced as described above.

## Results and discussion

In this work 180 strains of lactic acid bacteria from different sources were evaluated for their ability to produce bioactive peptides with ACE-inhibitory and Ca-binding effects. The initial screening was made according to the total ACE-inhibitory and Ca-binding activities. In Table 1, 20 strains with the best ACE-inhibitory activity and their corresponding Ca-binding activity are shown. According to our results, the strains demonstrating the highest ACE-inhibitory activity possessed the best proteolytic activity, too. Among these strains the best producers of Ca-binding peptides were found. The two strains demonstrating the highest ACE-inhibitory activity showed the best Ca-binding activity. The strain with the strongest ACE inhibition was *L. helveticus* A1, and the same strain showed the second Ca-binding activity (6.08 mmol/L bonded Ca). This strain possesses good technological properties, such as fast acidification, production of aroma compounds and strong proteolytic activity. *L. helveticus* A1 is one of the best candidates for development of cheese starters. The strain showing the second best ACE-inhibitory effect was *L. casei* C3. This strain was the best producer of Ca-binding peptides. Another important result from the study of the total ACE-inhibitory activity was the registration of such activity for one *L. delbrueckii* subsp*. bulgaricus* strain (*L. bulgaricus* J24). It is a very rear case for a *L. delbrueckii* subsp*. bulgaricus* strain to demonstrate such a fair value of ACE inhibition.

**Table 1.** ACE-inhibitory and Ca-binding activities of the 20 strains with the highest activity.

No.	Strain	ACE-inhibitory activity (%)	Bonded Ca mmol/L (at 10 mmol/L total Ca)
1	*L. lactis* 1	58.4	1.88
2	*L*. *lactis* 3	3.1	0.21
3	*L*. *lactis* 4	3.0	0.15
4	*L*. *lactis* 6	7.2	0.1
5	*L*. *lactis* 7	9.0	0.04
6	*L*. *lactis* 10	8.7	0.09
7	*L*. *helveticus* 4	14.2	1.64
8	*L*. *helveticus* 7	12.2	2.08
9	*L*. *helveticus* 8	60.1	2.64
10	*L*. *plantarum* 2	13.2	0.2
11	*L*. *helveticus* A1	94.0	6.08
12	*L*. *casei* 1	7.8	2.8
13	*L*. *casei* C3	82.1	6.4
14	*L*. *casei* 4	38.2	4.08
15	*L*. *salivarius* 3	3.5	0.1
16	*L*. *helveticus* Hh	34.0	4.32
17	*L*. *helveticus* V28	68.2	4.96
18	*L*. *helveticus* Q40	11.8	2.56
19	*L*. *helveticus* 48	9.3	3.48
20	*L. delbrueckii* subsp*. bulgaricus* J24	36.5	3.48

**Figure 1.**
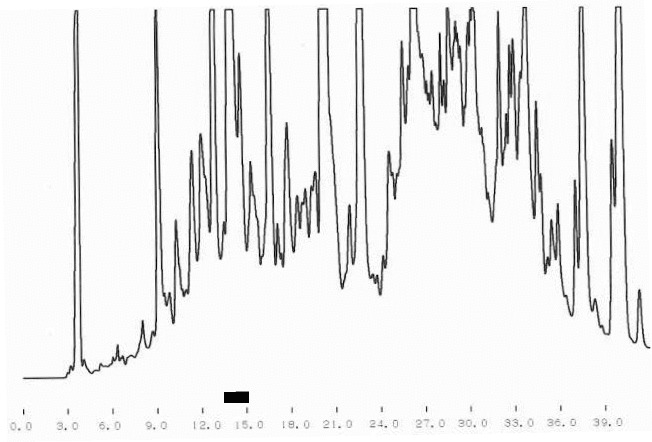
Crude fractionating of peptides released by strain *L. helveticus* A1.

**Figure 2.**
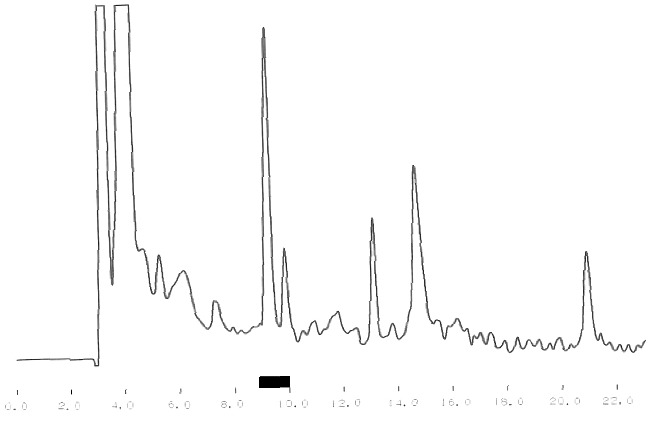
Reverse phase re-fractionating of the most active fraction received after the crude fractionating.

**Figure 3.**
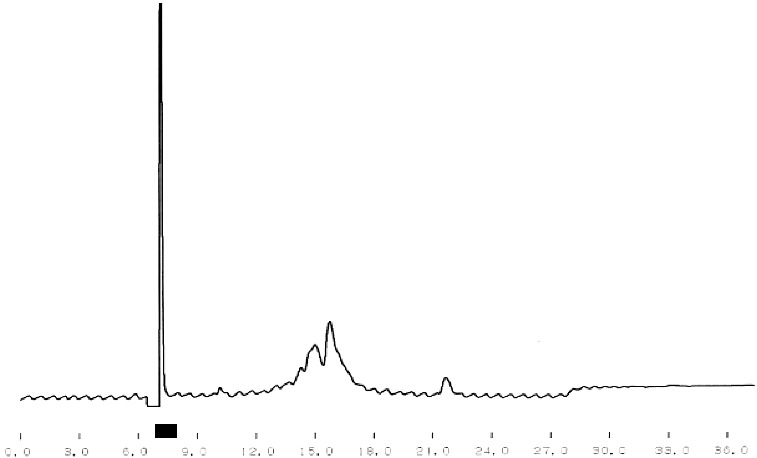
Ion-exchange purification of single ACE-inhibitory peptide from *L. helveticus* A1.

One of the main tasks of this study was the purification of bioactive peptides with the best ACE-inhibitory and Ca-binding effects. Figure 1 shows the results from the crude separation of the initially purified peptides. The fraction with the best ACE-inhibitory activity is underlined. The same fraction was subjected to additional fine fractionating using again RP-HPLC separation but with a specially adjusted gradient program. The corresponding chromatogram is shown in Figure 2. The fraction with the strongest ACE-inhibitory effect is underlined, too. The final purification step of this fraction was ion-exchange using pH gradient. This different principle of separation, comparing with the reverse phase, helps to receive well-purified single peptides. The chromatogram after the ion-exchange separation is shown in Figure 3 and again the fraction (single peptide) is underlined. Single peptides with the strongest ACE-inhibitory effect were purified from the strains which demonstrated the strongest total ACE inhibition. The ACE-inhibitory activity of the single peptides was proved using the modified test (see ‘Materials and methods’ section).

The bioactive peptides with a Ca-binding activity and especially with an ACE-inhibitory activity are usually small peptides. In order to analyse the molecular structure of the bioactive peptides, the general Edman sequencing in solution is not suitable because the derivatizing reagent phenyl-isothiocianate cannot be separated by extraction from the small peptides. That is why we bonded these peptides onto the membrane through aryl-amine connection. In this case, the C-end remained connected during the sequencing steps. The sequences of the peptides with the strongest ACE-inhibitory and Ca-binding properties are given in Table 2. In the case of *L. helveticus* A1, the peptide was found to contain four amino acid residues, and in the case of *L. casei* C3, five amino acids. Concerning the peptides with a Ca-binding effect, as expected, they were shown to be mainly phospho-peptides. Three amino acid residues could be phosphorilated: serine, threonine and tyrosine. In this study, we found only phosphoserine to be involved in the Ca-binding peptides. The main source of bioactive peptides with Ca-binding activity in the present research was β-casein and αS1-casein. These two casein fractions are subjected to proteolytic attacks during ripening of the cheeses much more than other fractions. The released peptide from the strain *L. helveticus* A1 demonstrating the best Ca-binding activity was QMEAESpISpSpSpEE. The possible source of this peptide is f 59-70 from αS1-casein. The strain *L. casei* C3 showed the best Ca-binding activity due to the peptide SpLSpSpSpE, corresponding to f 15-20 from ß-casein.

**Table 2.** Sequences of peptides with ACE-inhibitory activity released by the selected strains *L. helveticus* A1, *L. casei* C3 and *L. delbrueckii* subsp*. bulgaricus* J24.

Strain	Sequence of ACE-inhibitory peptides
*L. helveticus* A1	Ala-Leu-Pro-Met
*L. casei* C3	Ala-Pro-Phe-Ala-Lys
*L. delbrueckii* subsp*. bulgaricus* J24	Leu-Gly-Pro-Val-Arg-Gly-Pro-Phe-Pro

The release of such small bioactive peptides as these with ACE-inhibitory and Ca-binding effects is in parallel with a strong proteolytic activity of the strains. The technological conditions, such as the ripening temperature and the duration of cheese maturation, are key factors in the breakdown of the casein fractions by proteolytic enzymes and for the release of a sufficient quantity of bioactive peptides. After pilot production of functional cheeses with the developed starters, the ACE-inhibitory and calcium-binding effects were confirmed during the cheese ripening. As a control, we used cheese produced with the starter b189, which has proven its excellent properties during many years of application. The addition of the two specially selected adjunct cultures into the cheese starters led to increased production of bioactive peptides in the cheese products.

## Conclusions

The ability of lactic acid bacteria to release bioactive peptides is strain specific and is dependent on the dairy-processing conditions. In this study, we found two strains with strong ACE-inhibitory and Ca-binding effects. The released peptides by the two strains were purified and sequenced. The addition of such strains into the cheese starters led to the release of bioactive peptides in cheese products. In this way it is possible to increase the functional properties of Bulgarian white brined cheese.
